# Clinical application of regional citrate anticoagulation for membrane-based therapeutic plasma exchange in children with liver failure

**DOI:** 10.3389/fped.2023.1206999

**Published:** 2023-10-19

**Authors:** Jun Hu, Chunxiao Wang, Ke Bai, Chengjun Liu

**Affiliations:** ^1^ Intensive Care Unit, Ministry of Education Key Laboratory of Children Development and Disorders, Children’s Hospital of Chongqing Medical University, Chongqing, China; ^2^ International Science and Technology Cooperation Base of Child Development and Critical Disorders, Chongqing Key Laboratory of Pediatrics, Chongqing, China

**Keywords:** regional citrate anticoagulation, plasma exchange, liver failure, children, citrate accumulation

## Abstract

**Background:**

Regional citrate anticoagulation (RCA) is being used more commonly in children for continuous renal replacement therapy. Few reports describe the application of membrane-based therapeutic plasma exchange (mTPE) with RCA in children with liver failure (LF).

**Aims:**

To explore the application of RCA-mTPE in children with LF.

**Methods:**

We retrospectively analyzed data from children with LF who underwent RCA-mTPE in the Children's Hospital of Chongqing Medical University's pediatric intensive care unit. We used the total to ionized calcium ratio (T/iCa) > 2.5 as the diagnostic criteria for citrate accumulation (CA). The patients were divided into two groups according to the occureence of CA at the end of RCA-mTPE (CA group: T/iCa > 2.5; NCA group: T/iCa ≤ 2.5). To evaluate the clinical safety and efficacy of RCA-mTPE, the following data from medical records were assessed and compared between groups: clinical characteristics, reasons for LF, RCA-mTPE parameters and duration, laboratory findings, and complications.

**Results:**

In total, 92 RCA-mTPE treatments were administered to 21 children with LF over 3.8 ± 0.9 h. The following mean values were determined: blood flow rate (QB) = 2.8 ml/kg/min, 4% sodium citrate dose/blood flow rate ratio (QCi/QB) = 1.1(QCi,ml/kg/h); plasma dose/body weight ratio(QP/BW) = 18.5 (QP, ml/kg/h); 10% calcium gluconate dose/blood flow rate ratio (QCa/QB) = 0.2(QCa, ml/kg/h). The mean concentration of iCa *in vitro* was 0.38 ± 0.07 mmol/L. Citrate accumulation was recorded after 34 (37%) treatments. Hypocalcemia occurred in 11 (12%) and 7 (7.6%) treatments, during and after mTPE, respectively. Three hypotensive and one convulsive events, related to hypocalcemia, and two clotting events occurred during RCA-mTPE. After RCA-mTPE, the patients' pH, HCO_3_^−^ and Na^+^ levels, and T/iCa were significantly increased and the total bilirubin (TB), conjugated bilirubin (DB), prothrombin time (PT), activated partial thromboplastin time (APTT), alanine aminotransferase (ALT), aspartate aminotransferase (AST),and ammonia levels were significantly decreased. The TB, DB, and lactic acid levels, before RCA-mTPE, were significantly higher in the CA group than in the NCA group, but there were no significance between the two groups in QB/BW, QCi/QB, and QP/BW, mTPE duration, and estimated amount of citrate metabolized.

**Conclusions:**

Children with LF undergoing RCA-mTPE are at risk of hypocalcemia. With proper protocol adjustment, however, RCA-mTPE can be used safely and effectively in these patients.

## Introduction

The human liver is a vital organ that performs important functions such as synthesis, metabolism, detoxification, immunity, and secretion. The significant reduction of its functions may manifest with conditions such as abnormal blood coagulation, hypoproteinemia, jaundice, and hyperammonemia. Liver failure (LF) can have severe clinical manifestations and result in death. For those with LF, various supportive therapies, such as therapeutic plasma exchange (TPE), molecular adsorbent recirculation, and single-pass albumin dialysis, have been developed as temporary alternatives or bridging treatments before liver transplantation or self-recovery. TPE is the most commonly used technique for acute LF and has been included in European guidelines (1) as a level I, grade 1 recommendation for the management of this condition. Although the value of TPE for acute LF remains controversial (1–3).

Pediatric acute LF is a rare disease with reported mortality rates of 24%–53% (4, 5). It is diagnosed when a patient who has never had liver disease shows biochemical evidence of hepatic injury and significant coagulopathy and encephalopathy (6). Pediatric acute LF is characterized pathologically by hepatic necrosis, hepatocyte destruction, and bile duct proliferation (7). Children with liver injury typically show coagulation dysfunction because the liver is vital for the synthesis of blood coagulation factors.

TPE has been employed for numerous indications over the past decade, including the management of autoimmune and rheumatological diseases, LF, sepsis, hematological disorders, and renal and neurological diseases, and for drug removal (8). It often necessitates anticoagulation, especially when fresh-frozen plasma (FFP) is used as a therapeutic solution. Since most anticoagulants are metabolized in the liver, patients with LF frequently have coagulation disorders, making this procedure challenging (9).

Systemic heparin administration is the most commonly used method for hemodialysis and membrane therapeutic plasma exchange (mTPE), due to its anticoagulatory effect, rapid onset, and low cost; however, it may increase the risk of bleeding (10). Generally, the initial and maintenance doses of heparin maintain an activated clotting time (ACT) or activated partial thromboplastin time (APTT) 1.5–2 times that of normal (11). As the child's coagulation function improves with continuous FFP infusion, the heparin dose should theoretically be increased to maintain appropriate anticoagulation (11, 12). However, it is extremely difficult to increase the dosage of heparin appropriately, and improper adjustment may increase the risk of bleeding or clotting (13).

Citrate inhibits the coagulation cascade by reducing the concentration of ionized calcium (iCa) in the filter via chelation. The filter removes a portion of the calcium citrate complexes, and the remainder enters the systemic circulation and is metabolized rapidly. A constant citrate concentration can theoretically maintain a constant ionized calcium (iCa) concentration, and the blood does not clot when the concentration of iCa is less than 0.33 mmol/L (14). Because FFP contains a stable citrate concentration, it is not necessary to continuously increase the amount of citrate to maintain an appropriate iCa concentration *in vitro*; in fact, the amount of citrate must be reduced. Thus, the anticoagulant effect of citrate in TPE may be more stable.

Citrate is used more commonly in centrifugal TPE than in membrane-based TPE (mTPE), due to the less risks of citrate accumulation and hypocalcemia caused by greater citrate removal (80% vs. 20%–30%) (15). mTPE is a common PE method that has the advantages of simple operation, low cost, and high separation capacity. Theoretically, these risks can be reduced by adjustment of the mTPE protocol to maintain the citrate dose within the patient's metabolic capacity. Few reports describe the application of mTPE with regional citrate anticoagulation (RCA-mTPE) in children with LF. The purpose of this study was to assess the efficacy, safety, and benefits of the RCA-mTPE protocol for children with LF, to provide a basis for the clinical selection of more optimal anticoagulation methods for this patient population.

## Methods

### Study design and patients

We conducted this retrospective study with data from children with LF who underwent RCA-mTPE at the Children's Hospital of Chongqing Medical University, Chongqing, China, between February 2020 and November 2021. The study was approved by the hospital's ethics committee [2022 NLS (Y) no. 146]. Written informed consent was obtained from all children's legal guardians. Pediatric acute LF(PALF) was diagnosed in accordance with the national diagnostic criteria for acute LF in children, including: (a) the absence of pre-existing liver disease, (b) severe LD suddenly occurred within 8 weeks of onset, and (c) uncorrectable coagulopathy (after the administration of vitamin K) with prothrombin time (PT) > 20 s and international normalized ratio (INR) > 2.0 in patients without hepatic encephalopathy, or coagulopathy with PT > 15 s or INR > 1.5 in patients with encephalopathy (9, 16).

#### Inclusion criteria

Meet all the following contents: (a) PALF diagnosis criteria were met and mTPE treatment was performed; (b) mTPE was anticoagulated with RCA.

#### Exclusion criteria

Meet any one of the following contents: (a) Despite received mTPE, PALF diagnostic criteria were not met; (b) mTPE was anticoagulated with other anticoagulation methods besides RCA.The patients were divided into two groups according to whether T/iCa > 2,5, which was often being used as a diagnostic criterion for citric acid accumulation(CA) (9), after RCA-mTPE (CA group: T/iCa > 2.5; NCA group: T/iCa ≤ 2.5).

### RCA-mTPE

RCA-mTPE was performed using the PlasautoΣ blood perfusion device. An appropriate membrane plasma separator was chosen according to each child's weight. When the extracorporeal circulation volume exceeded 10% of the blood volume or the patient had hypotension, a red blood cell suspension and albumin were used to prefill the extracorporeal circulation. Sodium citrate anticoagulant (136 mmol/L citrate; National Medicine Permit no. H20058913; Sichuan Nightingale Biological, China) was delivered to the arterial blood collection end of the device, and 10% calcium gluconate was delivered to the venous return end. The initial RCA-mTPE protocol is shown in Table 1. When the ratio of total to ionized calcium (T/iCa) exceeded 2.5 before RCA-mTPE, the initial blood flow rate, 4% sodium citrate dose, and plasma dose took the smaller value, while the 10% calcium gluconate dose took the larger value. FFP or normal plasma was anticoagulated using citrate dextrose solution II, resulting in a plasma citrate concentration of about 15 mmol/L.

**Table 1:** RCA-mTPE parameter settings.

Parameter	Initial parameters	Final parameters
QB, ml/kg/min	2–5	2.8
QCi/QB	0.8–1	1.1
QCa/QB	0.1–0.3	0.2
QP/BW	10–25	18.5

RCA-mTPE, regional citrate anticoagulation for membrane-based therapeutic plasma exchange.

QB, blood flow rate; QCi, 4% sodium citrate dose; QCa, 10% calcium gluconate dose; QP, plasma dose; BW, body weight.

For example, the initial parameters for a 10-kg child are: QB = 30 mL/min, QCi = 30 ml/h, QCa = 6 ml/h, and QP = 200 ml/h.

First, the sodium citrate dose was adjusted to attain an in-vitro iCa concentration of 0.2–0.4 mmol/L; it was increased above this range and reduced below it. The calcium gluconate dose was then adjusted to attain a target in-vivo iCa concentration of 1.0–1.35 mmol/L; it was reduced above this range and increased below it.

In-vivo and in-vitro blood gas analyses (ABL90 FLEX analyzer) were performed 30 min after the start of RCA-mTPE. Routine blood gas and component analyses were performed and biochemical indicators, electrolytes, and coagulation factors were evaluated after RCA-mTPE.

### Data collection and variable definitions

The following information was collected from the patients' medical records: reasons for LF, RCA-mTPE parameters and duration, laboratory findings, citrate accumulation (T/iCa > 2.5), hypocalcemia (iCa concentration < 0.9 mmol/L), hypercalcemia (iCa concentration > 1.35 mmol/L), hyponatremia (sodium ion concentration < 135 mmol/L), hypernatremia (sodium ion concentration > 145 mmol/L), metabolic acidosis (bicarbonate concentration < 22 mmol/L), and metabolic alkalosis (bicarbonate concentration > 27 mmol/L). When the waste liquid flow rate equaled the plasma dose + sodium citrate dose, the filter clearance rate was calculated as:

$$\mathrm{clearance}\;\mathrm{rate}=\frac{\mathrm{QP}+\mathrm{QCi}}{\mathrm{QB}\times 60+\mathrm{QCi}}.$$

where QP is the plasma dose, QCi is the sodium citrate dose, and QB is the blood flow rate. The amount of citrate metabolized by the body was estimated as:

Citratemetabolized(mmol/kg/h)=QCi×0.136×(1−filterclearancerate)+QP×0.015.

### Statistical analysis

The statistical analyses were performed with IBM SPSS (version 21). The chi-squared test and Fisher's exact test were used to assess differences in qualitative data. Continuous variables consistent with normal distribution were reported as mean ± standard deviation and compared using the *t* test, and without normal distribution were reported as median (first and third quartile) and compared using the Mann–Whitney *U* test. *P*-values <0.05 were considered to reflect significance.

## Results

### Patient characteristics

The sample comprised data from 21 children with LF who underwent 92 RCA-mTPE treatments with an average duration of 3.8 ± 0.9 h. The patients' clinical characteristics are summarized in Table 2.

**Table 2:** Patients’ clinical characteristics.

Parameter	Value
*N*	21
Sessions, *n*	92
Age, months	22.00 (8.04, 74.00)
Body weight, kg	11.50 (8.50, 20.00)
Male gender, male (*n*, %)	12.00 (57.14%)
PRISM Ⅲ score	8.86 ± 4.69
Child-Pugh score	13.00 (11.00,14.00)
In-ICU days, days	14.02 ± 7.05
In-hospital days, days	25.27 ± 11.43
In-hospital mortality (*n*, %)	3.00 (14.29%)
90-day mortality (*n*, %)	10 (47.60%)
Causes of ALF (*n*, %)	
Drug	2 (9.52%)
Poisoning	3 (14.29%)
Infection	3 (14.29%)
Immune	4 (19.05%)
Metabolic	5 (23.81%)
Biliary tract diseases	2 (9.52%)
Others	2 (9.52%)

### Effect of RCA-mTPE on the clinical indicators

After RCA-mTPE, the patients' total and conjugated bilirubin levels, APTT, aspartate and alanine aminotransferase levels improved significantly, however, the pH, bicarbonate levels, and T/iCa also increased significantly. The *in-vivo* iCa concentration decreased significantly in CA group and increased significantly in NCA group (Table 3).

**Table 3:** Biochemical indicators, electrolytes, and blood gas parameters before and after RCA-mTPE.

	Before PE	After PE	*P*-value
TB, μmol/L	227.90 ± 95.60	169.30 ± 83.10	<0.001
DB, μmol/L	85.80 ± 59.90	48.70 ± 37.70	<0.001
PT, s	33.90 ± 16.70	20.80 ± 9.00	<0.001
APTT, s	50.50 ± 16.80	43.10 ± 15.30	<0.001
AST, u/L	144.50 (53.25, 447.93)	72.10 (37.25, 183.50)	<0.001
ALT, u/L	138.50 (51.00, 343.00)	80.15 (35.75, 157.55)	<0.001
blood ammonia, mmol/L	78.90 ± 42.40	69.90 ± 33.40	0.003
blood lactate, mmol/L	3.10 ± 2.20	2.80 ± 1.40	0.242
PH	7.43 ± 0.07	7.47 ± 0.08	<0.001
HCO_3_^−^, mmol/L	25.50 ± 5.60	30.50 ± 5.10	<0.001
iCa^2+^, mmol/L	1.18 ± 0.11	1.14 ± 0.17	0.465
T/iCa	2.03 ± 0.27	2.48 ± 0.51	<0.001
Na^+^, mmol/L	137.10 ± 4.00	139.80 ± 3.60	<0.001
Effects of citric acid-induced fluid load			
Heart rate	117.37 ± 20.63	116.39 ± 21.79	0.639
Mean arterial pressure, mmHg	72.42 ± 16.00	78.10 ± 17.17	0.002
Diastolic blood pressure, mmHg	56.14 ± 12.81	60.52 ± 14.89	0.002
CA group			
PH	7.42 ± 0.08	7.44 ± 0.06	0.122
HCO_3_^−^, mmol/L	26.17 ± 6.04	28.72 ± 5.39	0.001
iCa^2+^, mmol/L	1.18 ± 0.11	1.05 ± 0.19	0.001
NCA group			
PH	7.43 ± 0.05	7.48 ± 0.08	<0.001
HCO_3_^−^, mmol/L	25.18 ± 5.30	31.50 ± 4.67	<0.001
iCa^2+^, mmol/L	1.18 ± 0.12	1.24 ± 0.12	0.003

TB, total bilirubin; DB, direct (conjugated) bilirubin; PT, prothrombin time; APTT, activated partial thromboplastin time; AST, aspartate aminotransferase; ALT, alanine aminotransferase; T/iCa, total to ionized calcium ratio.

### Effects of RCA-mTPE on the fluid load and cardiovascular system

The heart rate, diastolic blood pressure, and mean arterial pressure were used to assess the effect of a citrate-induced increase in the fluid load. RCA-mTPE induced significant increases in patients' diastolic blood pressure and mean arterial pressure, but did not affect the heart rate (Table 3).

### General data and complications

CA occured in 8 (38.1%) patients, for a total of 34 (36.9%) RCA-mTPE treatments. Before RCA-mTPE, the TB, DB, and lactate levels were significantly higher in the CA group than in the NCA group. The PRISM III score, Child–Pugh score, prothrombin time (PT), APTT, aspartate and alanine aminotransferase levels, ammonia level, blood flow rate/ and plasma dose/body weight ratios, sodium citrate dose/blood flow rate ratio, PE duration, and estimated amount of citrate metabolized did not differ between the two groups (Table 4).

**Table 4:** Characteristics according to the occurrence of citrate accumulation after RCA-mTPE.

Parameter	NCA group	CA group	*P*-value
Sessions, *N*	58	34	
90-day mortality (%)	5/12 (41.67%)	4/9 (44.44%)	0.623
Before RCA-MTPE			
PRISM Ⅲ score	12.90 ± 2.10	10.20 ± 2.90	0.117
Child-Pugh score	12.50 ± 2.10	11.40 ± 3.60	0.430
PT (s)	35.90 ± 17.40	48.40 ± 44.30	0.152
APTT, (s)	46.80 ± 15.10	59.50 ± 34.00	0.065
TB (μmol/L)	191.00 ± 79.10	256.40 ± 110.90	0.011
DB (μmol/L)	63.00 ± 55.20	92.90 ± 65.40	0.042
ALT (u/L)	138.50 (51.00, 310.90)	122.00 (42.50, 389.00)	0.952
AST, (u/L)	103.00 (53.00, 386.73)	204.50 (54.00, 523.20)	0.363
Lactic acid (mmol/L)	2.10 ± 1.00	3.70 ± 2.60	0.003
Serum ammonia (mmol/L)	68.00 ± 40.00	84.00 ± 40.00	0.123
Final parameters			
QB/BW, (ml/min)	2.94 ± 0.38	2.89 ± 0.53	0.698
QCi/QB	1.04 ± 0.17	1.07 ± 0.22	0.518
QCa/QB	0.20 ± 0.07	0.22 ± 0.10	0.366
QP/BW, (ml/h)	18.07 ± 3.92	20.00 ± 7.39	0.164
DCi (mmol/kg/h)	0.64 ± 0.10	0.66 ± 0.15	0.364
PE duration, (h)	3.67 ± 0.70	3.94 ± 1.11	0.237

NCA, no citrate accumulation (T/iCa < 2.5 at the end of RCA-mTPE); CA, citrate accumulation (T/iCa > 2.5 at the end of RCA-mTPE); PT, prothrombin time; APTT, activated partial thromboplastin time; TB, total bilirubin; DB, direct (conjugated) bilirubin; ALT, alanine aminotransferase; AST, aspartate aminotransferase; QB, blood flow rate; BW, body weight; QCi, 4% sodium citrate dose; QCa, 10% calcium gluconate dose; QP, plasma dose; DCi, amount of citrate metabolized by the body.

During and after treatment, there were 11 (12%) and 5 (5.4%) episodes of hypocalcemia occurred, respectively, among which there were three episodes of hypotension and one episode of seizure, related to hypocalcemia, occurred. After RCA-mTPE, the incidence of metabolic alkalosis was significantly increased, that of hyponatremia was significantly decreased, and those of hypercalcemia, hypocalcemia, hypernatremia, and bleeding complications did not differ. Two (2.2%) filter clotting events occurred (Table 5).

**Table 5:** Complications occurring before and after RCA-mTPE.

	Before mTPE	After mTPE	*p*
iCa^2+^ < 0.9 mmol.L^−1^ (*n*, %)	0 (0.00%)	5 (5.43%)	0.059
iCa^2+^ > 1.35 mmol.L^−1^ (*n*, %)	5 (5.43%)	9 (9.78%)	0.266
PH < 7.35 (*n*, %)	10 (10.87%)	6 (6.52%)	0.295
PH > 7.45 (*n*, %)	36 (39.13%)	58 (63.04%)	0.001
HCO_3_^−^ > 27 mmol.L^−1^ (*n*, %)	36 (39.13%)	70 (76.09%)	<0.001
HCO_3_^−^ < 22 mmol.L^−1^ (*n*, %)	29 (31.52%)	5 (5.43%)	<0.001
Na^+^ > 145 mmol.L^−1^ (*n*, %)	6 (6.52%)	5 (5.43%)	0.756
Na^+^ < 135 mmol.L^−1^ (*n*, %)	17 (18.48%)	6 (6.52%)	0.014
T/iCa > 2.5 (*n*, %)	5 (5.43%)	33 (35.87%)	<0.001
Bleeding (*n*, %)	6 (6.50%)	5 (5.40%)	0.756
Clotting (*n*, %)	2(2.20%)		

iCa^2+^, *in-vivo* ionized calcium level; T/iCa, ratio of total to ionized calcium *in vivo*.

## Discussion

Although RCA-TPE has been in clinical use for more than a decade (17), few reports describe RCA-mTPE (8, 18–22), and even fewer provide specific RCA recommendations for mTPE for children with LF. In this study, we evaluated the clinical application of RCA-mTPE in 21 children with LF; the findings indicate that this treatment is safe and effective for this patient population when performed with strict monitoring and adjustment.

RCA-mTPE may be a credible anticoagulation option for children with LF, many of whom have coagulation disorders. Systemic heparin administration for anticoagulation in these patients results in significant prolongation of the APTT and ACT, increasing the risk of bleeding. The coagulation function of these patients improves gradually as FFP is exchanged, making it more challenging to adjust the heparin dose to maintain the desired integration target. The primary benefit of RCA is its ability to maintain effective anticoagulation in the extracorporeal circulation while having a minimal effect on coagulation *in vivo* (23). The commonly used RCA target in clinical practice is an in-vitro iCa concentration of 0.2–0.4 mmol/L (24–28). Numerous studies have shown that the maintenance of a constant citric acid concentration enables the maintenance of a relatively stable iCa concentration *in vitro*. Because normal plasma and FFP have the same citrate concentration (15 mmol/L), the flow rate of 4% sodium citrate does not need to be adjusted to maintain the anticoagulation target. As a result, RCA-mTPE may enable the maintenance of a more stable anticoagulant state than does the use of systemic heparin anticoagulation in children with LF. The risk of bleeding during TPE with citrate is significantly lower than that during TPE with heparin (0% vs. 11.8%) (8, 29). In our sample, the incidence of bleeding did not increase after RCA-mTPE and only two episodes (2.2%) of filter clotting, fewer than in other studies with RCA (3.0%–7.3%) (18, 19, 22) and SHA (4.1%) occurred (30).

RCA-mTPE may be feasibly performed in children with LF despite their reduced citrate metabolism. As citrate is metabolized predominantly in the liver, kidneys, and skeletal muscle, LF was historically regarded as a contraindication for this RCA. An increasing number of reports describes the use of RCA with continuous renal replacement therapy (CRRT) in patients with LF, including children (24, 31, 32). These studies have demonstrated that these patients have a diminished ability, rather than complete inability, to metabolize citrate. Thus, we argue RCA-mTPE can be used effectively and safely for children with LF with control of the citric acid dose within the range of the liver's metabolization ability.

Citrate accumulation is the most concerning complication for children with LF undergoing RCA-mTPE, as RCA, LF, and PE all contribute to its risk. T/iCa > 2.5 is used commonly as the diagnostic criterion for citrate accumulation. In this study, the rate of citrate accumulation and T/iCa values increased after RCA-mTPE (37.0% vs. 4.3% and 2.48 ± 0.51 vs. 2.03 ± 0.27, respectively). The percentage of children with at least one episode of citrate accumulation (38.1%) was significantly lower than those reported by Ma et al. (20) for adults who had undergone RCA-mTPE (67%) and by Keila et al. (33) (70%) for children with LF who had undergone RCA-CRRT. Citric acid is a non-toxic physiological organic acid associated with the primary risks of hypocalcemia and moderate metabolic acidosis (34). Thus, the evaluation of the safety of RCA based on the presence of these conditions may be more reasonable.

In theory, the occurrence of citrate accumulation in children with LF who have undergone RCA-mTPE is related primarily to the amount of citric acid that must be metabolized by the body and the individual's ability to metabolize citrate. In this study, the PRISM III score, Child–Pugh score, PT, APTT, ammonia level, blood flow rate/ and plasma dose/body weight ratios, sodium citrate dose/blood flow rate ratio, PE duration, and estimated amount of citrate metabolized did not differ between the CA and NCA group. However, the total and coagulated bilirubin and lactate concentrations were significantly higher in the CA group than in the NCA group. These findings demonstrate that the incidence of citrate accumulation may be related more closely to individuals' ability to metabolize citrate, when RCA-mTPE parameters are not significantly different. They suggest that children with elevated total and coagulated bilirubin and lactate levels who undergo RCA-mTPE are at increased risk of citrate accumulation.

Hypocalcemia is the most severe complication of RCA; in patients undergoing RCA-mTPE, it may be caused by citrate accumulation and insufficient calcium supplementation. When citrate accumulation occurs, reduced breakdown of calcium citrate can result in the reduction of iCa release. Furthermore, waste plasma contains iCa and calcium citrate, which cause more calcium to be lost than is provided by the replenished plasma, necessitating supplementation. Eleven (12.0%) and five (5.4%) hypocalcemia events occurred during and after treatment, respectively, in this study, fewer than reported in adults undergoing RCA-mTPE (16.6%–19.4%) (18, 22) and comparable to the 7% reported by Keila et al. (33) in children with LF undergoing RCA-CRRT. Three (3.2%) episodes of hypocalcemia-related hypotension, one (1.0%) episode of hypocalcemia-related seizure, and no arrhythmia occurred. These complications were relieved by single calcium supplements (10% calcium gluconate, 1 ml/kg). The incidence of hypocalcemia with related symptoms or signs was lower than that reported by Kissling et al. (35) (8.5%) in adult patients and comparable to that reported by Cortina et al. (36) (0.8%) in children undergoing RCA-mTPE.

In addition to citrate accumulation and hypocalcemia, RCA can cause other metabolic complications, such as acid–base and salt imbalances. Relative to that before treatment, the incidence of metabolic alkalosis was significantly higher (pH > 7.45, 63.0% vs. 39.1%, *p* = 0.001; bicarbonate concentration > 27 mmol/L, 76.1% vs*.* 39.1%, *p* < 0.001) and that of hyponatremia was significantly lower (18.5% vs*.* 6.5%, *p* = 0.01) after RCA-mTPE. These results reflect increases in the bicarbonate and sodium concentrations due to sodium citrate metabolism. However, RCA-mTPE did not alter the incidences of hypercalcemia, hypocalcemia, or hypernatremia.

Additionally, RCA-mTPE can cause fluid overload. On average, about 9 ml/kg sodium citrate (3 ml/kg/h for 3 h) was used throughout the RCA-mTPE period. The increase in fluid overload resulted in significant increases in the diastolic blood pressure and mean arterial pressure, but did not affect the heart rate. We eliminated the excess fluid with diuresis or subsequent CRRT.

RCA is an effective PE modality for children with LF. The average in-vitro iCa concentration was 0.38 ± 0.07 mmol/L and the average duration of RCA-mTPE was 3.8 ± 0.9 h, with clotting occurring in two (2.1%) sessions. The total and coagulated bilirubin levels, alanine and aspartate aminotransferase levels, PT, and APTT were significantly lower after than before treatment, indicating that the treatment was effective.

The use of different RCA-mTPE parameter settings can result in a variety of complications. The blood flow rate, sodium citrate dose, and plasma dose collectively determine the amount of citrate that enters and needs to be metabolized in the patient's body. In this study, the T/iCa value was significantly higher after than before treatment (2.48 ± 0.51 vs. 2.03 ± 0.27, *p *< 0.001). Parameters such as the blood flow rate, sodium citrate dose/blood flow rate ratio, and plasma dose/body weight ratio can be reduced to reduce the risk of citrate accumulation.

### Limitations

This study is limited by its retrospective nature, which prevented us from establishing causal relationships; we could only examine associations. Furthermore, as individual patients could have received RCA-mTPE, RCA-CRRT, and/or FFP infusions, the pretreatment laboratory indicators may not accurately reflect the patients' liver function or citrate metabolism capacity, thereby influencing the analysis of citrate accumulation.

## Conclusions

This study demonstrated that RCA-mTPE is a safe and effective treatment for LF in children. In children undergoing RCA-mTPE, the risk of citrate accumulation is related most to the liver's capacity for citrate metabolism, which decreases more with increasing total and coagulated bilirubin and lactate levels, than with increasing aspartate and alanine aminotransferase and ammonia levels. The risk of citrate accumulation can be reduced by reducing parameters such as the blood flow rate, sodium citrate dose/blood flow rate ratio, and plasma dose/body weight ratio, which makes RCA-mTPE safer for children with LF.

## Data Availability

The original contributions presented in the study are included in the article/Supplementary Material, further inquiries can be directed to the corresponding author.

## References

[1] Wendon J Cordoba J Dhawan A Larsen FS Manns M Nevens F EASL clinical practical guidelines on the management of acute (fulminant) liver failure. J Hepatol. (2017) 66:1047–81. 10.1016/j.jhep.2016.12.003 28417882

[2] Tan EX-X Wang M-X Pang J Lee G-H. Plasma exchange in patients with acute and acute-on-chronic liver failure: a systematic review. WJG. (2020) 26:219–45. 10.3748/wjg.v26.i2.219 31988586 PMC6962432

[3] Padmanabhan A Connelly-Smith L Aqui N Balogun RA Klingel R Meyer E Guidelines on the use of therapeutic apheresis in clinical practice—evidence-based approach from the writing committee of the American society for apheresis: the eighth special issue. J Clin Apher. (2019) 34:171–354. 10.1002/jca.21705 31180581

[4] Rajanayagam J Coman D Cartwright D Lewindon PJ. Pediatric acute liver failure: etiology, outcomes, and the role of serial pediatric end-stage liver disease scores. Pediatr Transplant. (2013) 17:362–8. 10.1111/petr.12083 23586473

[5] Kathemann S Bechmann LP Sowa J-P Manka P Dechêne A Gerner P Etiology, outcome and prognostic factors of childhood acute liver failure in a German single center. Ann Hepatol. (2015) 14:722–8. 10.1016/S1665-2681(19)30767-7 26256901

[6] Bhatt H Rao GS. Management of acute liver failure: a pediatric perspective. Curr Pediatr Rep. (2018) 6:246–57. 10.1007/s40124-018-0174-7 32288972 PMC7102106

[7] Lefkowitch JH. The pathology of acute liver failure. Adv Anat Pathol. (2016) 23:144–58. 10.1097/PAP.0000000000000112 27058243

[8] Yuan F Li Z Li X Liu H. Application of regional citrate anticoagulation in membrane therapeutic plasma exchange. Int Urol Nephrol. (2020) 52:2379–84. 10.1007/s11255-020-02581-0 32740788

[9] Hu F Sun Y Bai K Liu C. Clinical application of regional citrate anticoagulation for continuous renal replacement therapy in children with liver injury. Front Pediatr. (2022) 10:847443. 10.3389/fped.2022.847443 36304531 PMC9592741

[10] Kindgen-Milles D Brandenburger T Dimski T. Regional citrate anticoagulation for continuous renal replacement therapy. Curr Opin Crit Care. (2018) 24:450–4. 10.1097/MCC.0000000000000547 30247214

[11] Smythe MA Priziola J Dobesh PP Wirth D Cuker A Wittkowsky AK. Guidance for the practical management of the heparin anticoagulants in the treatment of venous thromboembolism. J Thromb Thrombolysis. (2016) 41:165–86. 10.1007/s11239-015-1315-2 26780745 PMC4715846

[12] Hirsh J Anand SS Halperin JL Fuster V. Guide to anticoagulant therapy: Heparin.

[13] Kitchen S. Problems in laboratory monitoring of heparin dosage: review. Br J Haematol. (2000) 111:397–406. 10.1111/j.1365-2141.2000.02308.x 11122078

[14] James MFM Roche AM. Dose-response relationship between plasma ionized calcium concentration and thrombelastography. J Cardiothorac Vasc Anesth. (2004) 18:581–6. 10.1053/j.jvca.2004.07.016 15578468

[15] Ahmed S Kaplan A. Therapeutic plasma exchange using membrane plasma separation. CJASN. (2020) 15:1364–70. 10.2215/CJN.12501019 32312791 PMC7480555

[16] Di Giorgio A D’Antiga L. Acute liver failure in children: is it time to revise the diagnostic criteria? Liver Transpl. (2020) 26:184–6. 10.1002/lt.25693 31778017

[17] Antonič M Gubenšek J Buturović-Ponikvar J Ponikvar R. Comparison of citrate anticoagulation during plasma exchange with different replacement solutions. Ther Apher Dial. (2009) 13:322–6. 10.1111/j.1744-9987.2009.00733.x 19695068

[18] Christiadi D Mercado C Singer R. Regional citrate anticoagulation in membrane based plasma exchange: safety, efficacy and effect on calcium balance: citrate anticoagulated plasma exchange. Nephrology. (2018) 23:744–7. 10.1111/nep.13088 28618127

[19] Betz C Buettner S Geiger H Jung O. Regional citrate anticoagulation in therapeutic plasma exchange with fresh frozen plasma—a modified protocol. Int J Artif Organs. (2013) 36:803–11. 10.5301/ijao.5000245 24338655

[20] Ma Y Chen F Xu Y Wang M Zhou T Lu J Safety and efficacy of regional citrate anticoagulation during plasma adsorption plus plasma exchange therapy for patients with acute-on-chronic liver failure: a pilot study. Blood Purif. (2019) 48:223–32. 10.1159/000500408 31216551

[21] Sigler K Lee J Srivaths P. Regional citrate anticoagulation with calcium replacement in pediatric apheresis. J Clin Apher. (2018) 33:274–7. 10.1002/jca.21594 29027706

[22] Halpin MR Chen B Singer RF. Efficacy, safety, and calcium balance of an accelerated citrate anticoagulated membrane-based plasma exchange algorithm. Blood Purif. (2022) 51:70–4. 10.1159/000515827 33975318

[23] Chegondi M Vijayakumar N Totapally BR. Management of anticoagulation during extracorporeal membrane oxygenation in children. Pediatr Rep. (2022) 14:320–32. 10.3390/pediatric14030039 35894028 PMC9326610

[24] Soltysiak J Warzywoda A Kociński B Ostalska-Nowicka D Benedyk A Silska-Dittmar M Citrate anticoagulation for continuous renal replacement therapy in small children. Pediatr Nephrol. (2014) 29:469–75. 10.1007/s00467-013-2690-6 24337319 PMC3913856

[25] Cortina G McRae R Chiletti R Butt W. The effect of patient- and treatment-related factors on circuit lifespan during continuous renal replacement therapy in critically ill children. Pediatr Crit Care Med. (2020) 21:578–85. 10.1097/PCC.0000000000002305 32343111

[26] Levi M Opal SM. Coagulation abnormalities in critically ill patients. In: O’Donnell JM Nácul FE, editors. Surgical intensive care medicine. Cham: Springer International Publishing (2006). p. 222. 10.1186/cc4975 PMC1750988 16879728

[27] Sık G Demirbuga A Annayev A Citak A. Regional citrate versus systemic heparin anticoagulation for continuous renal replacement therapy in critically ill children. Int J Artif Organs. (2020) 43:234–41. 10.1177/0391398819893382 31856634

[28] Raymakers-Janssen PAMA Lilien M van Kessel IA Veldhoen ES Wösten-van Asperen RM van Gestel JPJ. Citrate versus heparin anticoagulation in continuous renal replacement therapy in small children. Pediatr Nephrol. (2017) 32:1971–8. 10.1007/s00467-017-3694-4 28578542 PMC5579151

[29] Soares Ferreira Júnior A Hodulik K Barton KD Onwuemene OA. Hemostatic effects of therapeutic plasma exchange: a concise review. J of Clinical Apheresis. (2022) 37:292–312. 10.1002/jca.21973 35196407

[30] Jiao J Yu Y Wei S Tian X Yang X Feng S Heparin anticoagulation versus regional citrate anticoagulation for membrane therapeutic plasma exchange in patients with increased bleeding risk. Renal Fail. (2023) 45:2210691. 10.1080/0886022X.2023.2210691 PMC10187106 37183868

[31] Brophy PD Somers MJG Baum MA Symons JM McAfee N Fortenberry JD Multi-centre evaluation of anticoagulation in patients receiving continuous renal replacement therapy (CRRT). Nephrol Dial Transplant. (2005) 20:1416–21. 10.1093/ndt/gfh817 15855212

[32] Klingele M Stadler T Fliser D Speer T Groesdonk HV Raddatz A. Long-term continuous renal replacement therapy and anticoagulation with citrate in critically ill patients with severe liver dysfunction. Crit Care. (2017) 21:294. 10.1186/s13054-017-1870-3 29187232 PMC5707786

[33] Rodriguez K Srivaths PR Tal L Watson MN Riley AA Himes RW Regional citrate anticoagulation for continuous renal replacement therapy in pediatric patients with liver failure. PLoS One. (2017) 12:e0182134. 10.1371/journal.pone.0182134 28792509 PMC5549692

[34] Schneider AG Journois D Rimmelé T. Complications of regional citrate anticoagulation: accumulation or overload? Crit Care. (2017) 21:281. 10.1186/s13054-017-1880-1 29151020 PMC5694623

[35] Kissling S Legallais C Pruijm M Teta D Vogt B Burnier M A new prescription model for regional citrate anticoagulation in therapeutic plasma exchanges. BMC Nephrol. (2017) 18:81. 10.1186/s12882-017-0494-9 28249613 PMC5333425

[36] Cortina G McRae R Chiletti R Butt W. Therapeutic plasma exchange in critically ill children requiring intensive care. Pediatr Crit Care Med. (2018) 19:e97–e104. 10.1097/PCC.0000000000001400 29401139

